# KLF4 Promotes Angiogenesis by Activating VEGF Signaling in Human Retinal Microvascular Endothelial Cells

**DOI:** 10.1371/journal.pone.0130341

**Published:** 2015-06-15

**Authors:** Yinan Wang, Chuanhe Yang, Qingqing Gu, Michelle Sims, Weiwang Gu, Lawrence M. Pfeffer, Junming Yue

**Affiliations:** 1 Department of Pathology and Laboratory Medicine, University of Tennessee Health Science Center, Memphis, Tennessee, United States of America; 2 Center for Cancer Research, University of Tennessee Health Science Center, Memphis, Tennessee, United States of America; 3 Department of Laboratory Animal Center, Southern Medical University, Guangzhou, P. R. China; University of Illinois at Chicago, UNITED STATES

## Abstract

The transcription factor Krüppel-like factor 4 (KLF4) has been implicated in regulating cell proliferation, migration and differentiation in a variety of human cells and is one of four factors required for the induction of pluripotent stem cell reprogramming. However, its role has not been addressed in ocular neovascular diseases. This study investigated the role of KLF4 in angiogenesis and underlying molecular mechanisms in human retinal microvascular endothelial cells (HRMECs). The functional role of KLF4 in HRMECs was determined following lentiviral vector mediated inducible expression and shRNA knockdown of KLF4. Inducible expression of KLF4 promotes cell proliferation, migration and tube formation. In contrast, silencing KLF4 inhibits cell proliferation, migration, tube formation and induces apoptosis in HRMECs. KLF4 promotes angiogenesis by transcriptionally activating VEGF expression, thus activating the VEGF signaling pathway in HRMECs.

## Introduction

Angiogenesis is the physiological process of forming new blood vessels from preexisting vasculature. While angiogenesis is required during growth and development, abnormal angiogenesis is associated with human disease including cancer, cardiovascular diseases, diabetes, age-related macular degeneration (AMD), and diabetic retinopathy (DR) [1–4]. Thus, modulators of angiogenesis have been used to treat these diseases. The vascular endothelial growth factor (VEGF) pathway promotes angiogenesis [5–8], and VEGF antibody and VEGF receptor (VEGFR) kinase inhibitors such as pegaptanib sodium, ranibizumab and bevacizumab have been approved for clinical use to treat cancer, AMD and DR [7, 9, 10]. Although these approaches have showed clinical efficacy, some side effects have been reported, which include cutaneous lupus erythematosus and intraocular central nervous system (CNS) lymphoma [11–13]. Therefore, further study is needed to understand the regulation of VEGF signaling pathways in order to develop more efficacious treatments with minimal side effects.

The Krüppel-like factors (KLFs) are a family of Zinc finger DNA binding transcriptional factors, which includes 18 KLFs and 9 specificity proteins (SP1s) [14, 15]. Only a few KLF family members have been studied in vascular endothelial cells. KLF2, KLF5, KLF6, KLF10 and KLF15 have all been shown to promote angiogenesis under various conditions [16–19]. In addition, KLF4 has been linked to tumor metastasis through regulation of the epithelial mesenchymal transition (EMT) in several forms of human cancers [20–23]. EMT promotes angiogenesis by targeting the VEGF pathway [24–27]. KLF4 is a key regulator in maintaining endothelial progenitor cell phenotypes and is upregulated by the leukemia inhibitory factor (LIF) and vascular endothelial growth factor (VEGF) through activation of the AKT pathway [28]. While KLF4 was reported to impair tube formation in endothelial cells by inhibiting cyclinD1 through upregulation of miR-15a [29], in another study KLF4 was reported to promote angiogenesis [30].

Human umbilical vein endothelial cells (HUVECs) and human retinal microvascular endothelial cells (HRMECs) differ in a number of properties as well as in their gene expression profiles [31]. While the role of KLF family members, including KLF4, in HUVECs has been investigated, their role in HRMECs has not been investigated. In addition it is unclear whether KLF4 contributes to the ocular neovascularization by regulating the VEGF pathway. In our present studies, we determined the role of KLF4 in HRMECs following lentiviral overexpression or knockdown of KLF4. We provide the first experimental evidence that KLF4 is a proangiogenic regulator of the VEGF signaling pathway in HRMECs.

## Materials and Methods

*Cell culture-* Primary human retinal microvascular endothelial cells (HRMECs) were purchased from Cell Systems (Kirkland, WA) and cultured in Medium 131 (Life Technologies, Grand Island, NY) supplemented with 10% FBS (Hyclone, Logan, Utah), as well as in 1% Microvascular Growth Supplement, 10μg/ml gentamicin, 100 U/ml penicillin, and 100 μg/ml streptomycin (Life Technologies, Grand Island, NY) at 37°C in a humidified 5% CO_2_ incubator. All experiments were performed on HRMECs within the first five passages.

### Lentiviral Vector Production

KLF4 and EGFP doxycycline (Dox)-inducible and reverse transactivator (rtTA-M3) lentiviral vectors were constructed using standard molecular cloning procedures as described previously [23]. The KLF4 shRNA lentiviral vectors KLF4shR1, TRCN0000005316 and KLF4shR2, TRCN0000010934 were purchased from GE Dharmacon (Lafayette, CO). Targeting sequences for KLF4 shRNA were 5’ GCTCCATTACCAAGAGCTCAT and (KLF4shR1) and 5’GCCAGAATTGGACCCGGTGTA (KLF4shR2). Scramble control, pLKO1-scramble (#1864) was purchased from Addgene (Cambridge, MA). Lentivirus was packaged in HEK293FT cells and produced as described previously[32]. HRMECs were transduced with lentivirus at 10 MOI, and pools of transduced cells were selected with 3 μg/ml puromycin.

### MTT assay

5000 cells were plated in 10% serum containing media into 96-well plates. After 8 h incubation, cells were serum-starved in 1%FBS for 12h and then treated with VEGF (50ng/ml). At various times, 10 μl of MTT reagent was added to each well and the plates incubated for approximately 4 h. The reaction was terminated by adding 100 μl of detergent, and plates incubated at 22°C in the dark for 2 h. Cell proliferation was calculated by measuring the absorbance (OD) at 570 nm wavelength.

### Cell migration assay

The transwell migration assay was performed using modified chambers (BD Sciences, Franklin Lakes, NJ) inserted into 24-well plates. 2 × 10^4^ cells in 300μl serum-free M131 medium were added into the upper chamber. VEGF was diluted in M131 medium to a final concentration of 50 ng/ml and added to the lower chamber of each well as the chemoattractant. After 12 h incubation, the medium and unmigrated cells in the upper chamber were removed, and the migrated cells in the lower side of the membranes were fixed with methanol and stained with Crystal Violet. Images were taken using an inverted microscope. Migrated cells were counted from at least three different fields and migration rate was calculated by normalizing to vehicle treated control cells.

### Tube formation assay

Matrigel (BD Biosciences) was added to 96-well plates (60 μl/well) for polymerization by incubating for 30 min at 37°C. Serum-starved ECs were resuspended in M131 medium with or without 50 ng/ml VEGF and then plated on top of the matrigel for incubation overnight at 37°C in a 5% CO_2_ incubator. At least three representative fields from each well were selected to count the branch points of tube-like structures and the data normalized to vehicle treated controls.

### In Vivo matrigel plug assay

To assess the angiogenic effect KLF4 in vivo, two month old C57/BL6 mice (3 each group) were subcutaneously injected with KLF4 expressing or control cells mixed with 0.5ml matrigel, respectively. All animal studies were approved by the Institutional Animal Care and Use Committee of University of Tennessee Health Science Center. At 10 days after injection, mice were sacrificed and plugs were collected and crysectioned. The numbers of microblood vessels were counted in CD31-immunostained sections of plugs at high magnification (40x). At least 3 sections from each matrigel plug were counted, and results from all three matrigel plugs were averaged as described previously [33].

### Luciferase reporter gene assay

0.2- and 1.5-kb VEGF promoter luciferase reporter plasmids [34](kind gift from Dr. Debabrata Mukhopadhyay at Mayor Clinic, Minnesota) were co-transfected into HRMECs transduced with KLF4 Tet-on lentiviral vector with pSV40 Renilla plasmid, and then 1ug/ml Dox was added. Luciferase and Renilla activity was measured at 24h following transfection, and the results was expressed as the ratio of luciferase versus Renilla activity.

### Chromatin immunoprecipitation (ChIP)

ChIP was performed using Active Motif kit (Carlsbad, CA) according to manufacturer’s instructions. KLF4-expressing (Dox treated 48h) and control HRMECs (no Dox) were cross-linked with 1% formaldehyde and sonicated. Cell lysates were immunoprecipitated with KLF4 antibody or IgG (Santa Cruz Inc., Dallas, Texas). Purified chromatin DNA (4 μl) was used for quantitative PCR to detect the presence of VEGFA promoter with the primers listed in supplemental (SP) table 1. KLF4 binding activity to VEGFA prom1ter was shown by the chromatic enrichment of KLF4 versus IgG and then normalized to control cells as described previously [23].

### Cell apoptosis

HRMECs transduced with KLF4 and scrambled RNAs were harvested following 12h serum starvation and lysed at different time points following viral vector transduction. The supernatant was isolated by centrifugation and cell apoptosis was detected by the Cell Death Detection ELISA kit (Roche Diagnostics Corporation, Indianapolis, IN) according to manufacturer’s instructions.

### Detection of VEGF released in cell culture media

VEGF released into cell media in KLF4 expressing and control cells was detected by ELISA (Aviva Systems Biology, San Diego, CA) according to manufacturer’s instructions. To examine whether KLF4 rescues the inhibition of VEGF production in culture media, KLF4 expressing and control cells were transfected with 30nM VEGF siRNA and scramble siRNA (Santa Cruz Inc., Dallas, Texas) for 24h and then cells were trypsinzed and counted and then 5x10^4^ cells were seeded uplayer of transwell to examine cell migration. Migrated cells were stained Crystal Violet and counted following 12h incubation.

### Immunofluorescent staining

To detect the expression of VEGF and KLF4 in HRMECs, KLF4-expressing and control cells were fixed for 10 min using 4% PFA, washed three times with 0.1% Tween-20 in PBS (PBST), and incubated with blocking buffer (5% normal goat serum, 3% bovine serum albumin, and 0.1% Triton-X 100 in PBS) for 1 h. The fixed cells were incubated with primary antibodies against VEGF and KLF4 (1:200 dilution, Santa Cruz, Dallas, Texas), overnight. After rinsing three times for 5 min with PBST, cells were incubated with Alexa488- or Alexa594-conjugated goat anti-rabbit (1:200 dilution, Life Technologies) antibodies for 1 h at room temperature. Cell nuclei were counter-stained with DAPI (Vector Laboratories, Inc.; Burlingame, CA). Images were taken using a Nikon inverted fluorescence microscope.

### Western Blot

*-* HRMECs transduced with KLF4 and control lentiviral vectors were serum- starved for 24h and then treated with 50 ng/ml VEGF, and cells were collected at different time points in RIPA buffer (Thermo Scientific) containing 1% Halt Proteinase inhibitor Cocktail (Thermo Scientific). Equal amounts of protein (30 μg/lane) were loaded on SDS-PAGE gels and then transferred onto nitrocellulose membranes. The membrane was blocked with 5% non-fat milk for 1 h and incubated with primary antibodies against KLF4 purchased from Santa Cruz (Dallas, TX), GAPDH from Sigma (St. Louis, MO), active caspase3, p-ERK, p-AKT, and p-VEGFR2 from Cell Signaling (Danvers, MA).

### Statistical Analysis

The significant differences were analyzed using a Student’s *t*-test. P < 0.05 was considered significant.

## Results

### Overexpression and knockdown of KLF4 in HRMECs using lentiviral vector

To investigate the function of KLF4 in HRMECs, KLF4 was overexpressed by Dox inducible [23] and silenced using shRNA lentiviral vectors, respectively. To induce KLF4 expression in HRMECs, Dox (1 μg/ml) was added to cell culture media for 24h. The expression of KLF4 was detected by Western blot using KLF4 antibody and showed approximately 6-fold increase as compared to cells not treated with Dox (S1A Fig). KLF4 was knocked down by lentiviral pLKO1 shRNA vector: two different shRNA vectors targeting different sequences of KLF4 gene were used. The knockdown effect following transduction and selection with puromycin was examined by Western blot. KLF4 expression in HRMECs transduced with KLF4shR1 and KLF4shR2 was reduced ~86% and ~91% as compared to scrambled transduced control cells, respectively. Our results indicated that KLF4 expression was significantly silenced with both shRNA knockdown vectors compared to controls (S1B Fig).

### KLF4 promotes basal and VEGF-induced cell proliferation, while silencing KLF4 results in apoptosis

**Fig 1:**
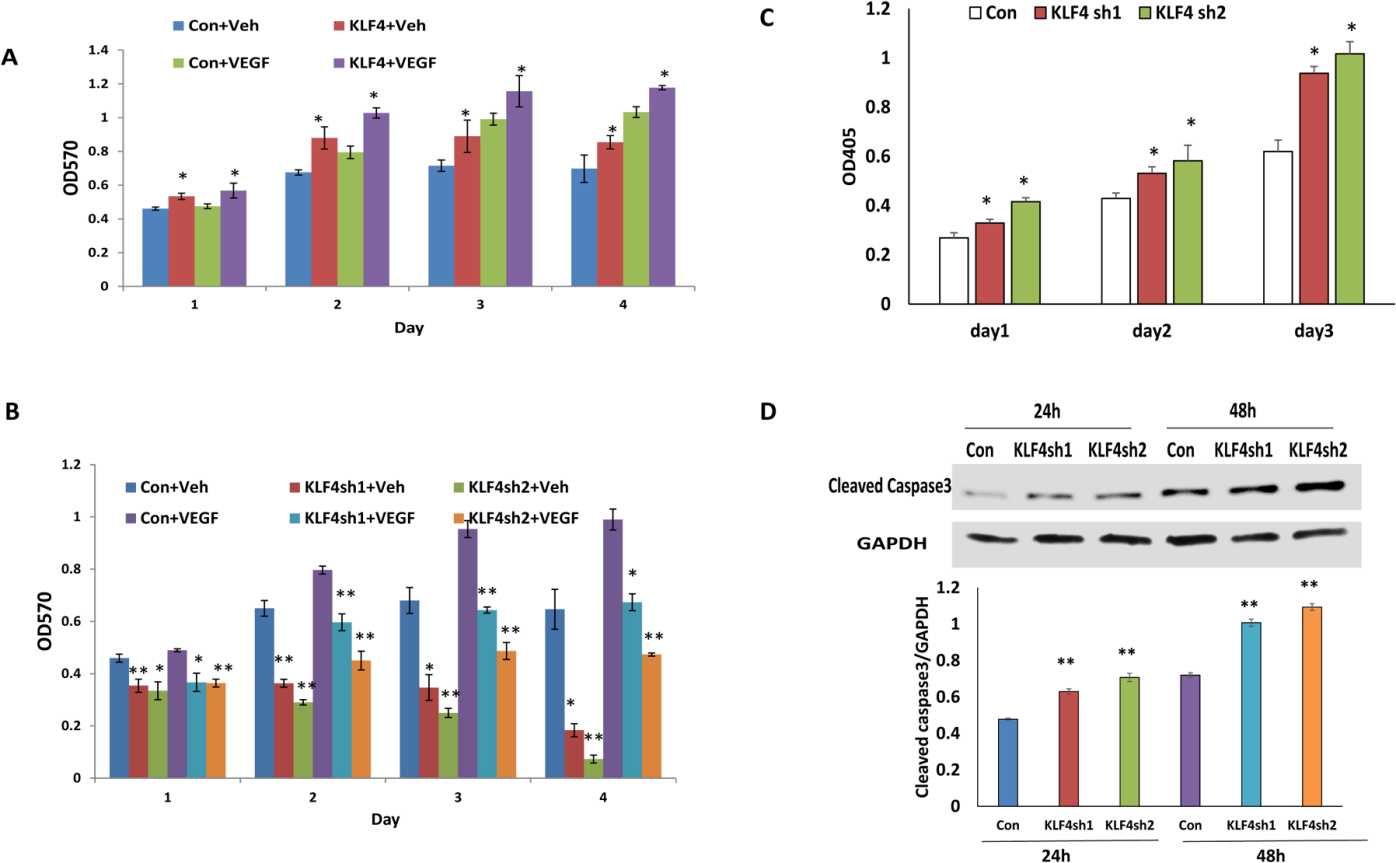
KLF4 promotes VEGF induced cell proliferation. **A, B.** Cell proliferation was examined at different time points in KLF4 expressing and knocking down HRMECs. Cells were treated with VEGF or vehicle (Veh) following 24h serum-free media culture before measuring cell proliferation using a MTT assay. Significance was observed between KLF4 expressing and control cells with or without VEGF induction. **C**. Cell apoptosis was examined using ELISA from HRMECs transduced with lentiviral KLF4shRNA1, 2 and Scramble controls. **D.** One representative Western blot was shown on the active caspase3 expression at indicated time points in KLF4 knockdown and control cells following 12h serum starvation. Significance was compared between KLF4 knockdown and control cells at the indicated time points. Data are presented as mean ±S.E. from 3 independent experiments, (*p<0.05, **p<0.01).

To examine the effect of KLF4 on VEGF induced-HRMEC proliferation, MTT assays were performed on KLF4-overexpressing and knockdown cells following 50 ng/ml VEGF treatment of serum-starved cells. Proliferation of KLF4-overexpressing cells (with Dox) was significantly increased as compared to control cells (not treated with Dox) at 24, 48, 72 and 96 h with or without VEGF treatment (Fig 1A). In KLF4 shRNA1- and shRNA2-transduced cells, cell proliferation was significantly reduced as compared to empty vector-transduced control cells at 24, 48, 72 and 96 h with or without VEGF treatment (Fig 1B). To test the effect of KLF4 on apoptosis, we examined apoptosis in HRMECs transduced with either lentiviral KLF4 shRNA1, KLF4 shRNA2 or scrambled control vector using an ELISA-based assay. As shown in Fig 1C, silencing KLF4 expression with either targeting vector significantly increased cell apoptosis at 24h and 48h culture. Cell apoptosis was also verified by the expression of active caspase3 as shown by Western blot (Fig 1D).

### KLF4 promotes VEGF-induced migration and tube formation in HRMECs and enhances angiogenesis in vivo

**Fig 2:**
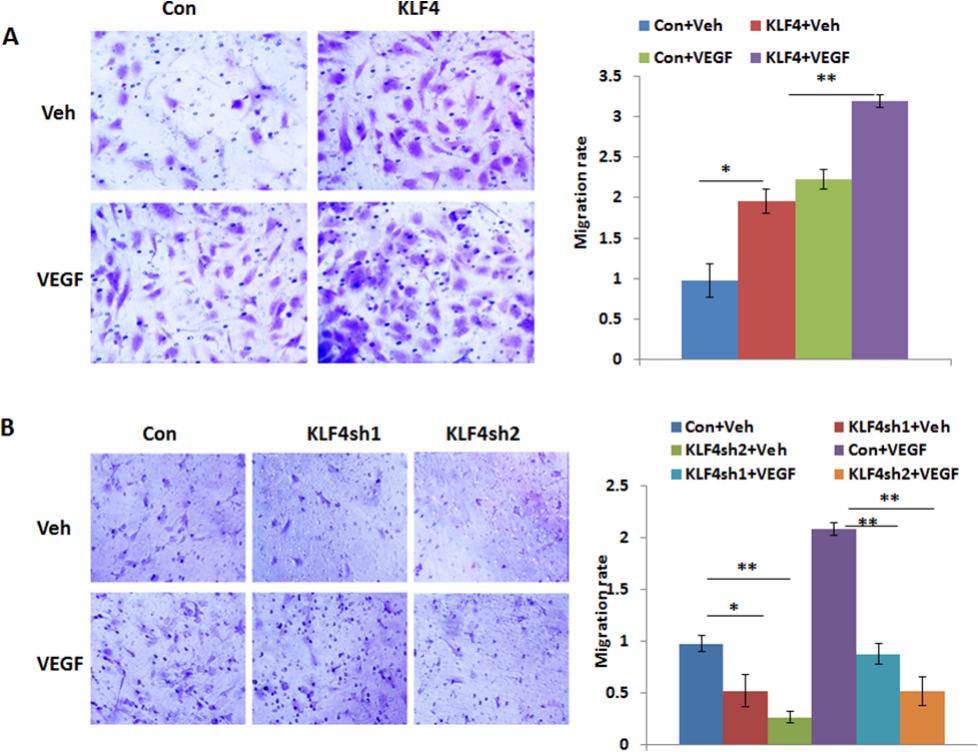
KLF4 promotes VEGF induced cell migration. **A, B.** Transwell migration assays were performed in HRMECs following KLF4 expression or knockdown. Migrated cells were stained with Crystal Violet and then counted. KLF4 expression in HRMECs significantly increased cell migration while knockdown of KLF4 reduced it as compared to control cells with and without VEGF treatment. The data were presented from three independent experiments in triplicate as mean+S.E and normalized by comparing it to vehicle treated control cells (*p < 0.05, **p<0.01).

**Fig 3:**
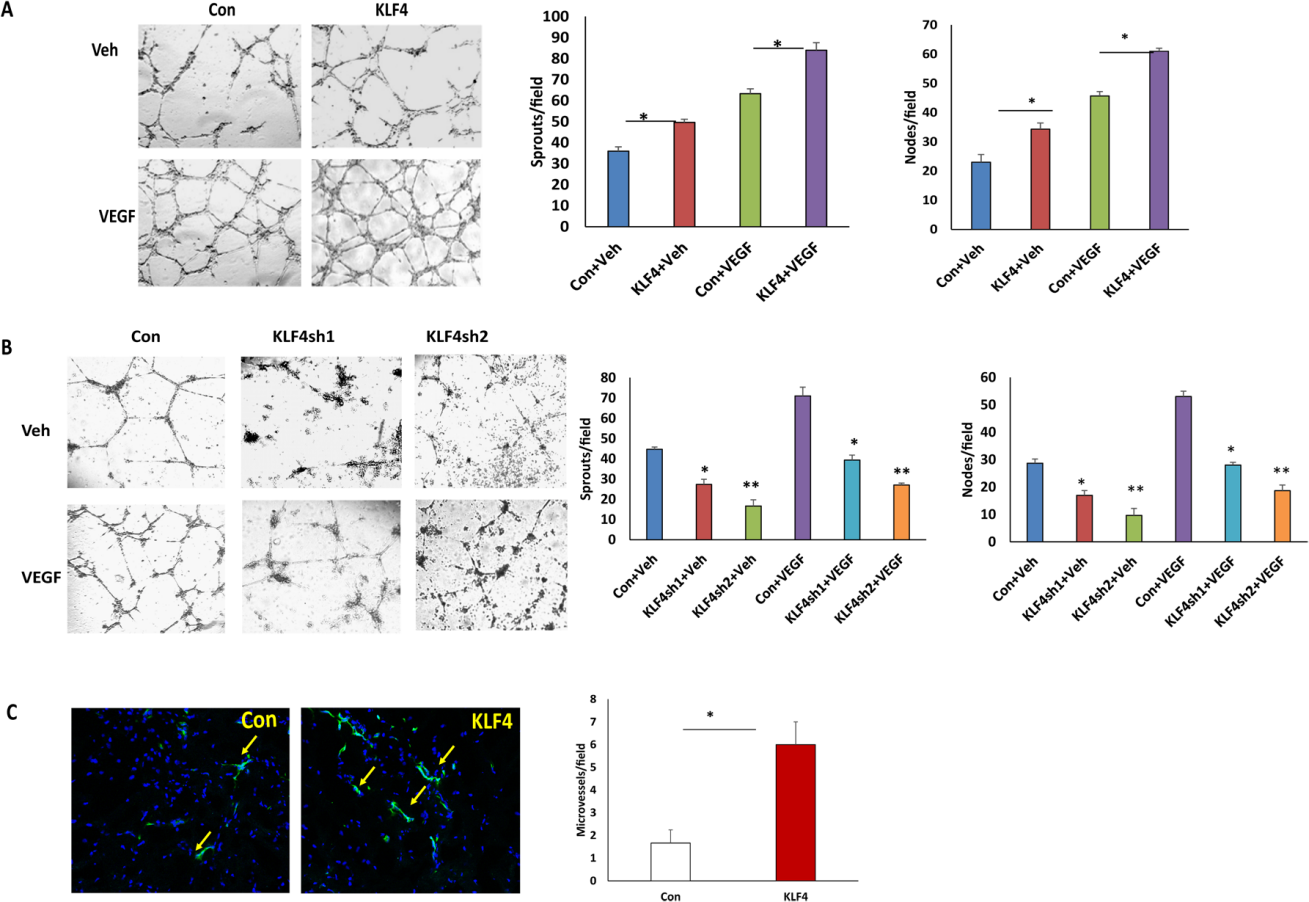
KLF4 promotes VEGF-induced tube formation and enhances angiogenesis in vivo. **A.B.** Tube formation assays were performed in KLF4 expressing and knockdown HRMECs, respectively. The angiogenic effect of KLF4 on VEGF induced tube formation was determined by counting nodes and sprouts of tube-like structures from at least three different fields of three independent experiments and normalized to vehicle treated control cells. Significance was compared between KLF4 expressing and control cells with or without VEGF treatment (*p<0.05, **p<0.01, ***p<0.001). Images were presented from one representative experiment. C. Sections of plugs were stained using CD31 antibody and microvessels were counted from 4 sections of each plug and averaged from total 3 plugs. Significance of CD31 positive vessels were compared between sections of KLF4 expressing and control plugs (*p<0.05).

To determine whether KLF4 affects migration in HRMECs, we performed transwell migration assays on HRMECs transduced with lentiviral KLF4-overexpression and shRNA-knockdown vectors using VEGF (50ng/ml) as chemoattractant. As shown in Fig 2A, overexpression of KLF4 significantly increased cell migration compared to control cells. In contrast, silencing KLF4 expression using two different KLF4 lentiviral shRNAs significantly reduced cell migration with or without VEGF (Fig 2B). To test how KLF4 affects VEGF induced angiogenesis, we then performed tube formation assays using both KLF4-overexpression and KLF4-knockdown cells. We found that overexpression of KLF4 significantly promotes VEGF-induced tube formation, while silencing KLF4 inhibits VEGF-induced tube formation in HRMECs (Fig 3A and 3B). To examine the angiogenic effect of KLF4 in vivo, we performed matrigel plug assays in mice using KLF4-expressing and control cells. Microvessels were identified by CD31 immunostaining on plug sections. CD31 positive stained microvessels were significantly higher in KLF4 expressing plugs than controls, indicating that KLF4 promotes angiogenesis (Fig 3C).

### KLF4 enhanced VEGF mediated signaling pathway in HRMECs

**Fig 4:**
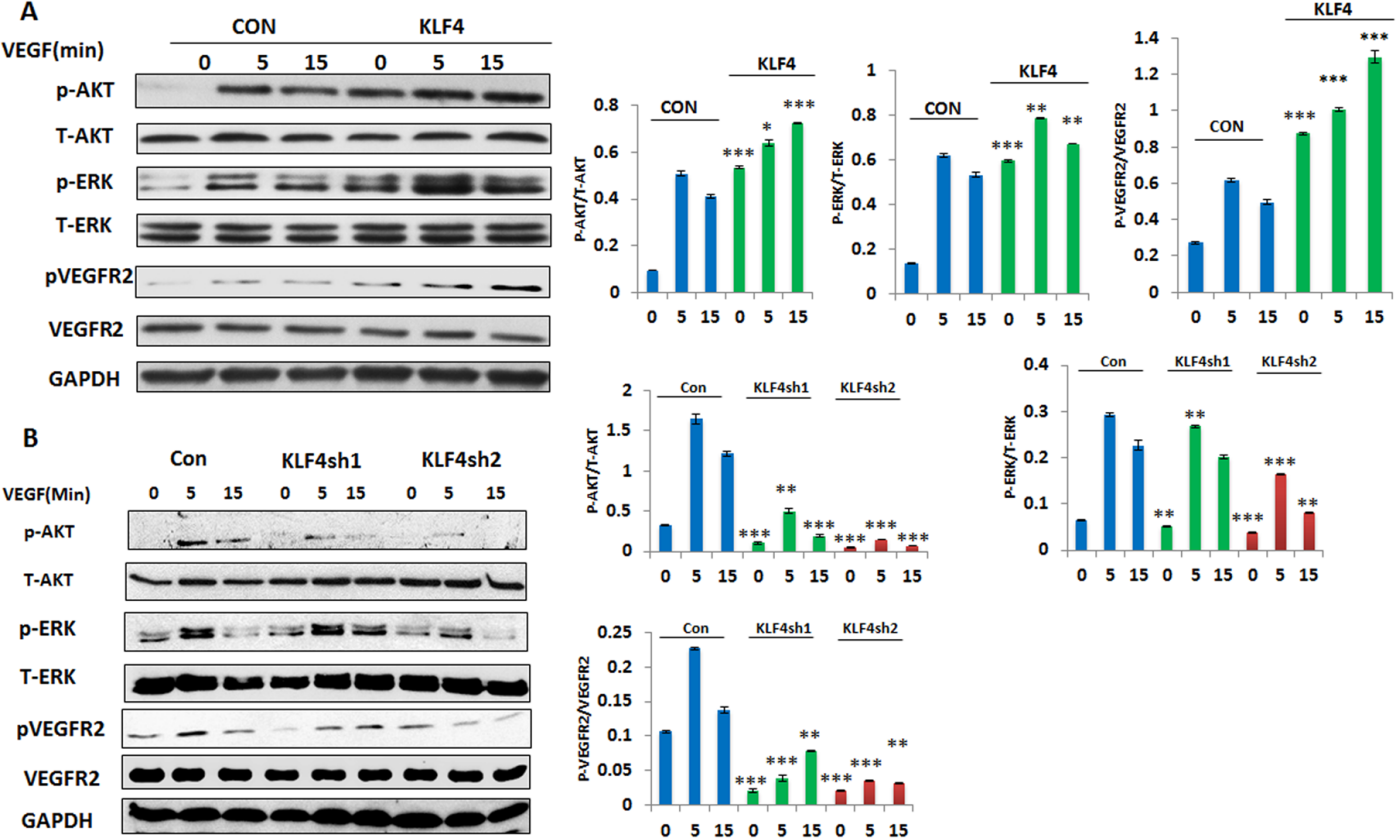
KLF4 enhances VEGF mediated angiogenesis signaling pathway. **A.B.** VEGF signaling pathway was examined in KLF4 expressing and knockdown HRMECs following serum starvation and VEGF treatment using Western blot. Data were presented from three separate experiments by measuring band density using the Image J program. Western blot was shown from one representative experiment. Significance was compared between KLF4 expressing and control cells at different time points of VEGF treatment (*p<0.05, **p<0.01, ***p<0.001).

The VEGF signaling pathway plays a key role in angiogenesis, and targeting the VEGF pathway by blocking the interaction between VEGF and its multiple receptors has been a focus in treating cancer, AMD, and proliferative DR. To understand how KLF4 functions in HRMECs through the VEGF signaling pathway, we treated KLF4 overexpression and knockdown cells with VEGF (50 ng/ml) for 0, 5 and 15 min following serum starvation, and then examined activation of VEGF signaling pathway as determined by phosphorylation of theVEGFR2 receptor subunit and two downstream cellular survival pathways ERK1/2 and AKT. We found that overexpression of KLF4 enhanced phosphorylation of VEGFR2 as well as VEGF-activated AKT and ERK1/2. In contrast, silencing KLF4 by two different KLF4 shRNAs attenuates the VEGF signaling pathway in HRMECs (Fig 4A and 4B). Our findings indicate that KLF4 activates VEGF signaling pathway in HRMECs.

### KLF4 transcriptionally activates VEGF expression

**Fig 5:**
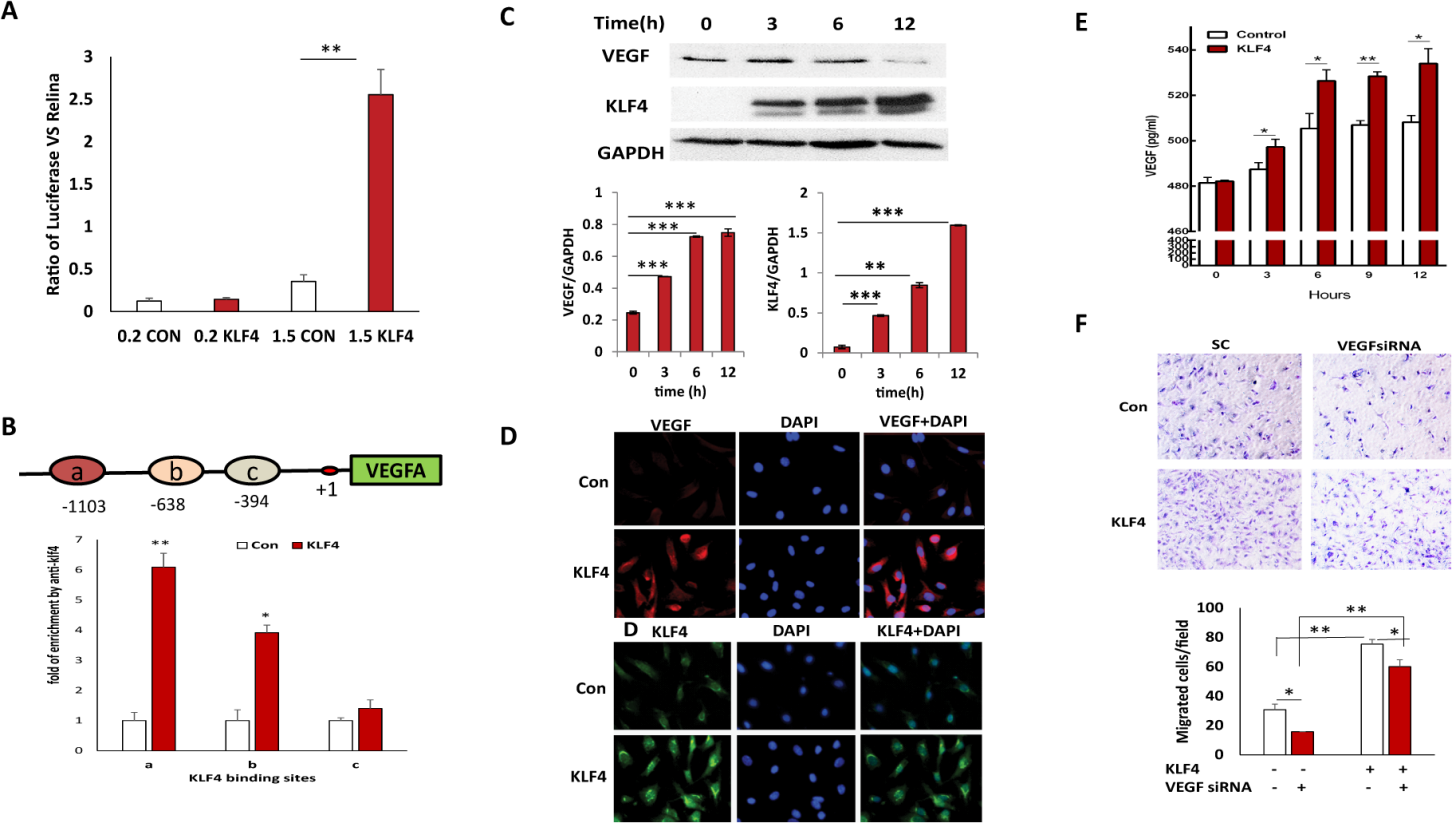
KLF4 transcriptionally activates VEGF expression. **A.** Luciferase reporter assays were performed to assess KLF4 activation of the VEGF promoter. Luciferase activities in KLF4 expressing and control HRMECs were measured at 24h after transfection with 1.5kb and 0.2kbVEGF promoter luciferase constructs in serum-free conditions. Data were presented as the mean ± SE from three independent experiments. Significance of luciferase activity was found between KLF4 expressing and control cells when 1.5Kb VEGF promoter was transfected in both cells (**p<0.01). **B.** There are three predicted KLF4 binding sites (CACCC) at the VEGFA promoter. The specific binding sites of KLF4 at the VEGFA promoter was detected by ChIP assay and enrichment of KLF4 binding to sites a and b of VEGFA promoter was significant, not c in KLF4 expressing compared to control cells (*p<0.05,**p<0.01) C: VEGF expression in HRMECs was detected by Western Blot at different time points. Significance was compared to 0h from 3, 6 and 12h (*p<0.05, **p<0.01, ***p<0.001). D: VEGF and KLF4 expression was imaged following immunofluorescence staining at 24h following KLF4 induction. E. VEGF released in cell media was detected using VEGF ELISA assay and significance was observed in KLF4 expressing compared to controls at the indicated time points(*p<0.05). F. Cell migration in KLF4 expressing and control HRMECs following VEGF knockdown using VEGF siRNA was examined using transwell migration assay. (*p<0.05, **p<0.01).

To characterize the molecular mechanism underlying the upregulation of VEGF signaling by KLF4, we examine the VEGF promoter for putative KLF4 binding sites and identified three potential binding sites (CACCC) within 1.5 kb upstream of the transcriptional initiation site. We examined whether KLF4 binds to the promoter of VEGF and activate the VEGF expression by transfecting HRMECs with two VEGF promoter-driven luciferase reporter constructs containing either 1.5 kb or 0.2 kb flanking sequences. While KLF4 binding sites are not found in the 0.2 kb VEGF promoter sequence, there are three potential binding sites within the 1.5 kb promoter region. Overexpression of KLF4 leads to significant upregulation of luciferase activity in HRMECs transfected with the 1.5 kb VEGF reporter vector. However, there are no significant differences in cells expressing the 0.2kb VEGF reporter plasmid as compared to control cells (Fig 5A). Our results indicate that KLF4 binds to the VEGF promoter between the 0.2 and 1.5 kb region and activates reporter gene luciferase expression. We have further defined the binding sites of KLF4 at the VEGFA promoter using ChIP assays and found that two predicted binding sites of KLF4 located at -638 and -1103bp from transcriptional start site at the VEGFA promoter were enriched approximately 4 and 6 fold in KLF4 expressing-cells as compared to control cells, respectively. These results indicate that both are bona fide binding sites for KLF4 to the VEGFA promoter and they drive VEGF transcription (Fig 5B).

**Fig 6:**
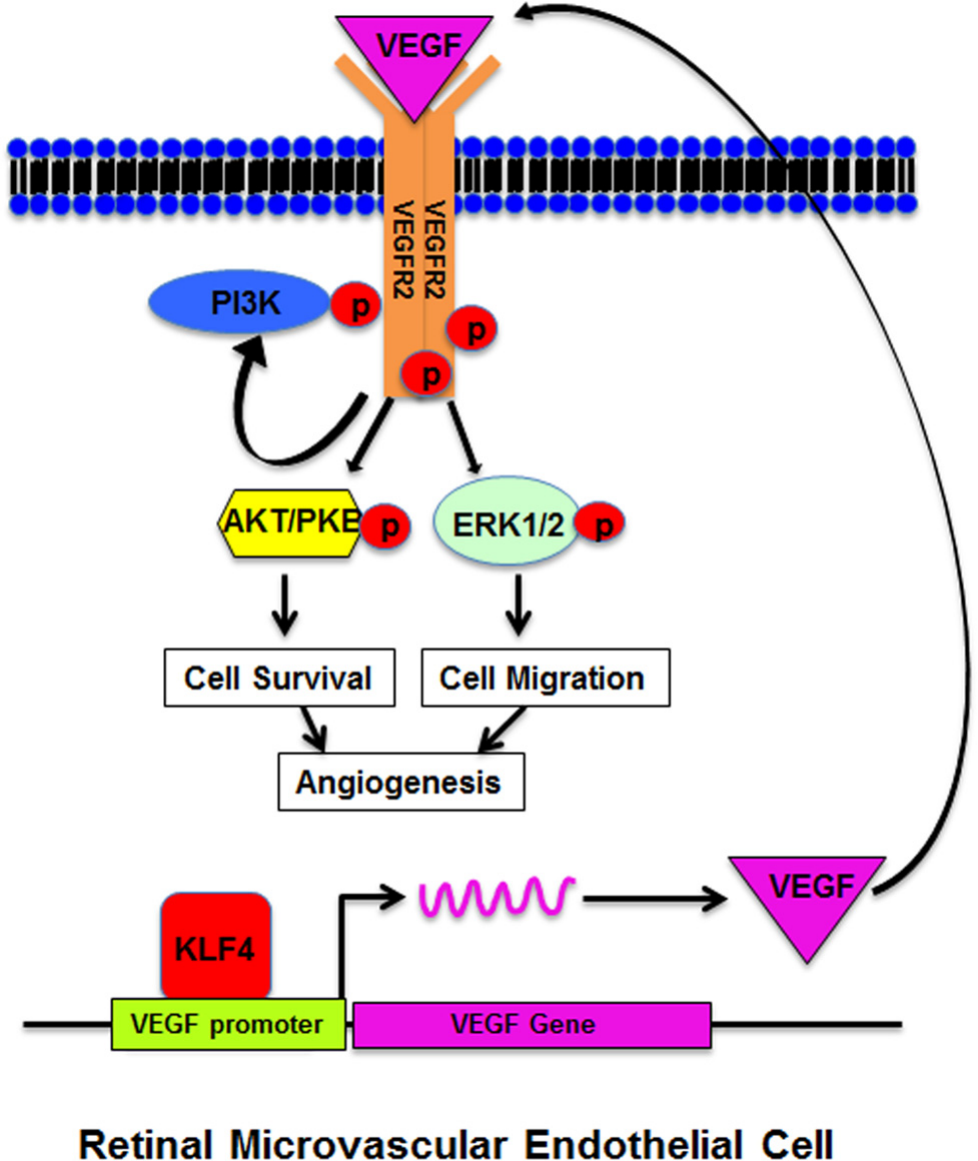
Schematic diagram of KLF4 mediated VEGF signaling pathway. KLF4 binds to the VEGF promoter and induces VEGF expression, subsequently phosphorylates VEGFR2 and activates downstream ERK1/2 and AKT to promote cell proliferation, migration and angiogenesis.

To further examine KLF4 regulation of VEGF expression, we induced KLF4 expression at different time points using Dox, and then examined VEGF expression in HRMECs by Western blotting. VEGF expression was significantly induced at 3, 6 and 12 h following the addition of Dox (Fig 5C). We also examined expression of VEGF and KLF4 by immunofluorescence staining. VEGF was stained in cytoplasm whereas KLF4 was stained in nuclei (Fig 5D). We further examined VEGF secreted in the supernatant using ELISA assay, and found that VEGF was significantly increased in KLF4-expressing cells as compared to control cells at the indicated time points (Fig 5E). Our rescue assay also indicated that KLF4 expression was able to rescue cell migration inhibited by knocking down the VEGF expression using VEGF siRNA (Fig 5F). Our results indicate KLF4 transcriptionally binds to VEGF promoter and induces VEGF expression, leading to VEGFR2 activation and downstream pERK1/2 and pAKT signaling to promote cell survival, migration and angiogenesis (Fig 6).

## Discussion

In our current studies, we have shown that KLF4 is a proangiogenic factor and promotes proliferation, migration and tube formation by enhancing the VEGF signaling pathway in HRMECs. Although KLF4 function has not been previously reported in HRMECs, a recent study showed that KLF4 promotes sprouting formation in HUVECs by targeting the Notch pathway [30]. It is also intriguing to note that sustained expression of KLF4 in endothelial cells leads to dysregulation of tumor sprouting angiogenesis and reduced tumor size [30]. In a separate study, KLF4 was shown to inhibit angiogenesis in HUVECs by activating miR-15a through transcriptional regulation [29]. It is unclear why these two studies had opposite results on the function of KLF4 in HUVECs, although two different mechanisms were proposed in defining how KLF4 regulates angiogenesis. Additional studies are needed to clarify the differences of the role of KLF4 in angiogenesis in HUVECs. In this study, we show for the first time that KLF4 promotes angiogenesis in HRMECs by utilizing both a highly efficient doxycycline inducible vector and a lentiviral knockdown strategy. Our findings are in agreement with those reported by Hale et al for the role of KLF4 in HUVECs [30] and suggest a similar function for KLF4 in both types of endothelial cells under certain conditions.

KLF4 has been shown to interact with KLF2 to maintain an intact endothelial layer of blood vessel and vascular integrity by upregulating the expression of downstream eNOS, VEGFR2, and occludin genes [35]. The VEGF signalling pathway contributes to the physiological and pathological conditions of angiogenesis. In our studies, we found that KLF4 is a positive regulator of the VEGF signalling pathway and thus promotes tube formation by activating the VEGF signalling in HRMECs. Previous studies have shown that KLF4 transcriptionally regulates several angiogenesis-associated genes, including forkhead box O1 (FOXO1), vascular endothelial growth factor (VEGF), and Kelch-like ECH-associated protein 1 (KEAP1) [36]. Therefore, we found that one molecular mechanisms by which KLF4 promotes angiogenesis in HRMECs is by transcriptional activation of the VEGF pathway, which was shown previous study [36]. We propose a novel mechanism whereby KLF4 promotes angiogenesis by transcriptionally activating the VEGF promoter, thus leading to VEGF expression, and subsequent enhancement of downstream pAKT and pEKR1/2 signaling in HRMECs.

KLF2 inhibits angiogenesis by downregulating VEGFR2 expression in HUVECs [37]. It is intriguing to note that KLF2 improved age-impaired neovascularization by promoting angiogenic cell survival through inducing eNOS expression [16]. This striking difference seems to be determined by the properties of mature EC and proangiogenic cells. We found that KLF4 promotes VEGF-induced VEGFR2 activation, while total VEGFR2 expression was not altered in HRMECs. Both KLF10 and KLF15 promote angiogenesis by binding and transcriptionally activating the promoters of Cox1 and bone morphogenetic protein (BMP) endothelial cell precursor-derived regulator (BMPER) in ECs, respectively. It is still unclear whether KLF4 also regulates Cox1 or BMP mediated signaling in ECs.

Together these studies indicate that the major mechanism underlying the role of the KLF family in endothelial cells is through transcriptional activation or repression of target genes, and subsequently activation or attenuation of downstream signaling pathways. There is a conserved binding site CACCC in the promoter of those genes regulated by KLF family members, such as the sites we identified in the VEGF promoter in this study and sites identified in COX1, BMPER and VEGFR2 throughout other reports[18, 19, 37, 38]. VEGF and VEGFR2 are directly involved in the VEGF signaling pathway, while BPMER participates in the TGFβ pathway. Both VEGF and TGFβ pathways are required for angiogenesis[39].

Because of the importance of the VEGF signalling pathway in angiogenesis, a focus on clinical therapy for human cancers, AMD, and DR and several other ocular neovascular diseases has been in development for inhibitors of VEGFR kinases to block the VEGF pathway. KLF4 is a positive regulator of VEGF signalling pathway as shown by our studies in HRMECs. Therefore, our finding on the roles of KLF4 in HRMECs will provide a novel therapeutic approach to regulate VEGF-induced angiogenesis through targeting KLF4 in treating ocular neovascular diseases.

## Supporting Information

S1 Fig Overexpression and Knockdown of KLF4 using lentiviral vectors A. **B.** KLF4 inducible expression and knockdown in HEMECs were detected by Western blot, respectively. The effect of KLF4 overexpression and knockdown was calculated from band intensity measured using image J (*p<0.05).
(TIF)

S1 Table Primer sequences used in Chromatin immunoprecipitation. (TIF)
