# Supplementary figures and images for: KLF4 Promotes Angiogenesis by Activating VEGF Signaling in Human Retinal Microvascular Endothelial Cells
Source: PLoS One. 2015 Jun 15;10(6):e0130341. doi: 10.1371/journal.pone.0130341 (PMC4467843; doi:10.1371/journal.pone.0130341)

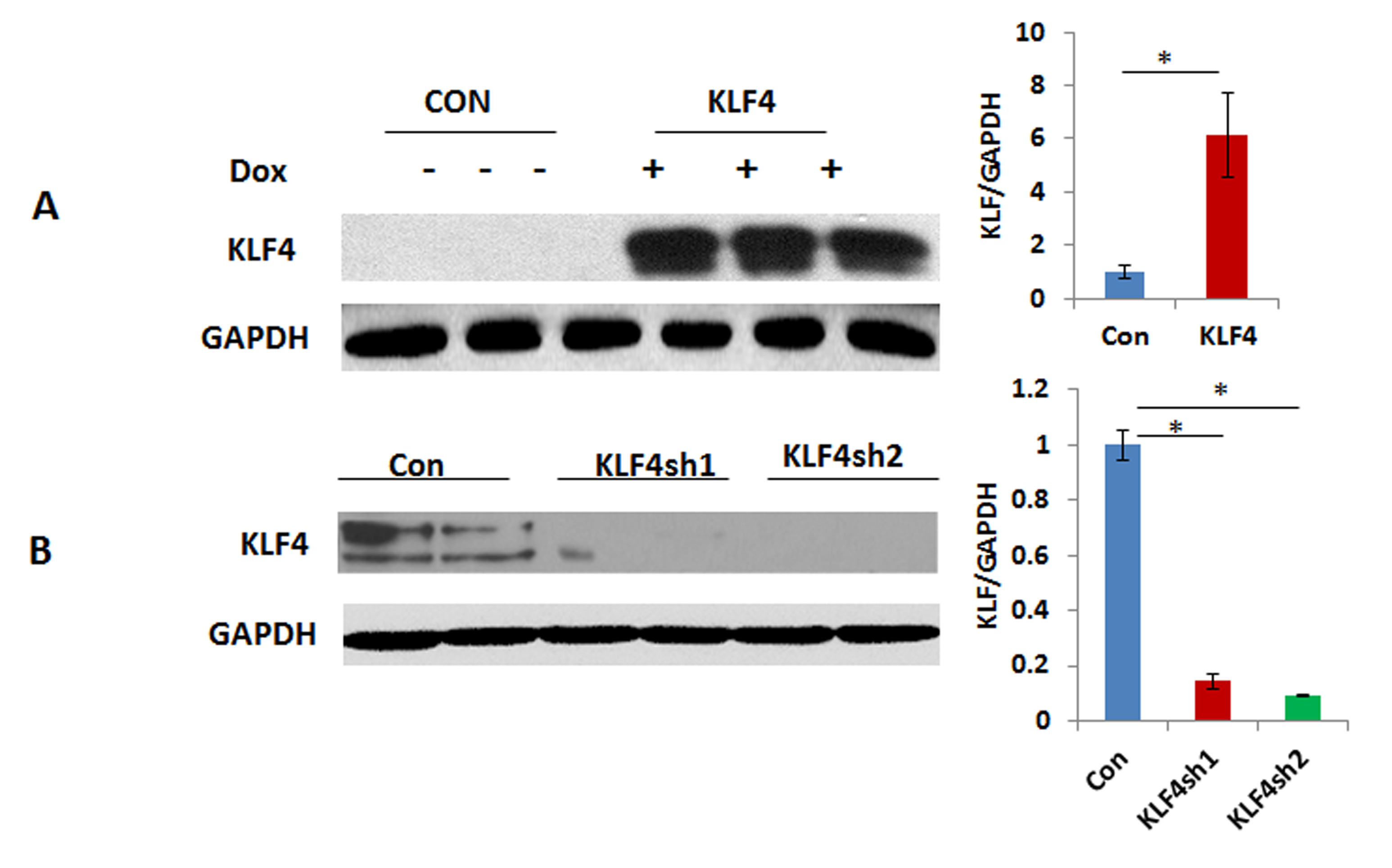

Supplement: S1 Fig — B. KLF4 inducible expression and knockdown in HEMECs were detected by Western blot, respectively. The effect of KLF4 overexpression and knockdown was calculated from band intensity measured using image J (*p<0.05). (TIF) [file pone.0130341.s001.tif]

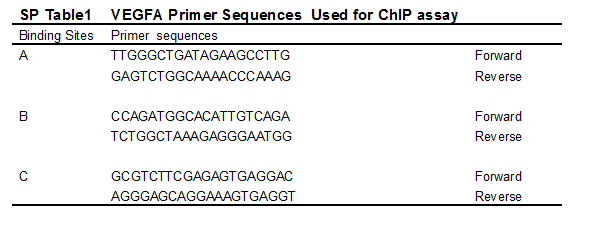

Supplement: S1 Table — (TIF) [file pone.0130341.s002.tif]
